# The weight concerns scale in Australian adolescents: Factor structure, gender invariance, and screening utility

**DOI:** 10.1007/s00787-026-03101-8

**Published:** 2026-06-22

**Authors:** Marcela Radunz, Maya Jabs, Daniel L. King, Tracey D. Wade

**Affiliations:** Discipline of Psychology, Flinders University, GPO Box 2100, Adelaide, South Australia 5001 Australia

**Keywords:** Factor structure, Weight concerns, Screening tool, Eating disorders, Adolescents

## Abstract

Despite the increased incidence of disordered eating among adolescents and low help-seeking rates, there is currently a lack of evidence supporting use of screening tools for this population. The present study aimed to address this gap by examining the factor structure, gender invariance, and psychological correlates of the Weight Concerns Scale (WCS) in a large sample of Australian adolescents to evaluate its potential suitability as a screening tool. Participants (*n* = 1022) aged 12–17 (M^age^ = 14.73; *SD* = 1.55) were recruited from five public secondary schools in South Australia between May and August 2023. All participants completed the WCS and psychological measures of life satisfaction, resilience, psychological distress, self-injurious thoughts and behaviours. Confirmatory factor analysis replicated the original one-factor structure: χ²(5) = 44.83, *p* < .001, RMSEA = 0.09, CFI = 0.99. Measurement non-invariance was found by gender and socio-economic-status, suggesting the scale functions differently across these subgroups. A cut-off score of ≥ 47 on WCS was associated with elevated psychological distress across all groups except for boys from lower socio-economic-status backgrounds. The present study is the first to apply confirmatory factor analysis to the WCS in adolescents. Future research needs to establish validity of the WCS as a screen in adolescents in terms of associations with ED psychopathology and behaviours. In addition, validation against diagnostic interview is crucial to establishing the WCS as a screening tool.

**Supplementary Information:**

The online version contains supplementary material available at https://doi.org/10.1007/s00787-026-03101-8.

## The weight concerns scale in Australian adolescents: Factor Structure, gender invariance, and screening utility

Around half of cases of feeding or eating disorders onset before age 18, with a peak age of emergence of 15.5 years [1]. This age group is particularly impacted by the increasing prevalence of EDs. Over the last four decades, the incidence of anorexia nervosa (AN) has been increasing significantly among girls aged 10–14, rising from 8.6 to 38.6 per 100,000 person-years [2]. In 2018, the prevalence of any self-reported DSM-5 ED in Australian adolescents was 22.2%, with rates of 12.8% in boys and 32.9% in girls [3]. A meta-analysis found that more than one in five children and adolescents in the community report disordered eating symptomology (22.36%; 95% CI 18.84%−26.09%; Lopez-Gil et al., [4]). Moreover, the COVID-19 pandemic exacerbated these trends, with the incidence of adolescent eating disorders increasing between 42% and 50% [5, 6] over this time. Data suggest that EDs increased more in this age group than depression or anxiety [5]. Additionally, there was an increase in the demand for ED-related care during the pandemic with increased inpatient admissions, hospital bed-days and outpatient care post-pandemic compared to pre-pandemic [7]. Since the pandemic, a population-based study has found a further increase in disordered eating in girls with a mean age of 15.8 years [8].

Many adolescents hide the core symptoms of their illness and delay help-seeking, resulting in EDs being underdiagnosed and undertreated [9, 10]. Meta-analyses have revealed that only one in four individuals with EDs seek help [11, 12]. Studies examining help-seeking behaviours in adolescents with eating disorders report lifetime help-seeking proportions ranging from 2.3% to 16.7% [10, 13–15]. Denial of illness (e.g., “*I don’t believe I have a problem”*) and ambivalence (e.g. “*I know my problems are serious*,* but I don’t want to lose them”*) are the most salient barriers to help-seeking and reflect the unique ego-syntonic nature of an ED [16]. These barriers may be intensified during adolescence, a period characterised by a strong push for independence and autonomy [17].

Given the increased incidence of disordered eating among adolescents and the low rates of help-seeking, it is crucial to have robust screening tools for EDs to improve early detection, and ideally, indicate some degree of severity such that people can be linked to an appropriate intervention (e.g., web-based psychoeducation or internet-CBT). Universal screening of anxiety and depressive symptoms conducted in this manner in Australian secondary schools resulted in a decreased risk of inhibited help-seeking and no mental-health related harms when compared to control [18]. However, a 2022 review of adolescent and adult screening tools conducted by the US Preventive Services Task Force concluded that the research evidence was inadequate to support the use of any screening tool for adolescents with eating disorders [19]. This review identified only three studies examining two different screening tools for adolescents Median age = 14–15 years), namely the Adolescent Binge Eating Disorder Questionnaire (ADO-BED; [20]) and the Sick, Control, One, Fat, Food (SCOFF; [21]). Other well-established and validated screening tools for eating disorders in adolescents such as the Eating Attitudes Test-26 (EAT-26; [22]) were deemed as unsuitable for use as a screener in primary care practice due to their length [19].

However, both the ADO-BED and the SCOFF have limited utility as comprehensive screening tools for the full spectrum of DSM-5 eating disorders in adolescents. The ADO-BED [20] tool was specifically designed to screen only adolescents with obesity and binge eating disorders (BEDs) in primary care. Thus, it was not designed to be a comprehensive screening tool for use across the wide range of eating disorders. On the other hand, the 5-item SCOFF [21], is the most widely used screening measure for eating disorders in adolescents, addressing AN, bulimia nervosa (BN) and BED. However, the US Preventive Services Task Force does not recommend the SCOFF for use with adolescents [19], reflecting limited evidence regarding its accuracy and clinical utility for this age group. The SCOFF primarily assesses overt behavioural symptoms (i.e., vomiting, rapid weight-loss), which may be less sensitive to the early, subthreshold disordered eating presentations that commonly emerge during adolescence and are more likely to present in community and primary care settings [3, 23].

The absence of a comprehensive, sensitive screening tool for adolescents represents a significant gap in current research and clinical practice. A more fruitful approach to screening and early identification in the context of early intervention for disordered eating in adolescents may be use of the Weight Concern Scale (WCS; [24]). This brief 5-item measure that focuses on cognitions and attitudes associated with weight and dieting has been shown to predict development of future EDs [25, 26], thus demonstrating its potential as a first-stage screening tool to identify elevated disordered eating risk and vulnerability in the community. The WCS was developed as part of a larger study [24] examining the effectiveness of a school-based intervention program design to prevent EDs in young adolescent girls (M^age^ = 12.7, *SD* = 0.72). Development was informed by the theoretical framework of the cognitive behavioural model of EDs, with items designed to measure the frequency and intensity of thoughts and behaviours related to weight and dieting. Killen et al., [24] used principal component analyses and found that weight concerns were highly associated with later onset of ED symptoms in their large sample of sixth and seventh-grade girls (*N* = 967) over a three-year period. In addition, the WCS has demonstrated excellent predictive validity, with many studies supporting the use of a cut-off score of 47 and above in predicting development of ED cases [25, 26].

The WCS has also been utilised as a screener for ED risk in samples of emerging adults as part of university-wide surveys, including the Healthy Minds Study [27], the German and Hungarian ProYouth programs [28, 29] and the Stanford-Washington University Eating Disorder Screen [30, 31]. However, to our knowledge, only two studies have used the WCS to screen for adolescents. Merwin and colleagues [32] screened a sample of 292 females aged 13–25 years, using a cut-off score of 52 to assess eligibility for digital acceptance and commitment therapy. Similarly, in a study examining the prevalence and correlates of EDs in Greek Cypriot youth, Koushiou et al., [33] used a WCS cut-off score of 52 to identify male and female adolescents and young adults (aged 13–32; M^age^ = 16.79; SD = 2.95; *n* = 1081) who were at low risk versus high risk for development of an ED.

Since its development, three studies have examined the factor structure of the WCS in young adults [34–36]. Dias and colleagues [36], in a sample of female university students in Brazil (*N* = 1084: M^age^ = 20.59, *SD* = 2.32), found an adequate fit (RMSEA = 0.08; CFI = 0.98). Similarly, da Silva and colleagues [35], examining 2068 female university students (M^age^ = 21.7, *SD* = 3.6) in Brazil and Mozambique, found good model fit in the former sample (RMSEA = 0.06, CFI = 0.99, TLI = 0.99) and excellent model fit in the latter sample (RMSEA = 0.02, CFI = 0.99, TLI = 0.99). Factor analyses of the combined sample (*N* = 2068) revealed good psychometric properties (RMSEA = 0.09, CFI = 0.98, TLI = 0.97).

Brasil et al. [34] are the only group to examine the gender invariance of the WCS using a large sample from the National Healthy Minds Study in the United States (*N* = 6981; M^age^ = 21.05, *SD* = 2.60). Findings revealed that the overall structure of the WCS was similar for men and women, demonstrating configural invariance. However, some differences emerged in how individual items functioned, such that men and women interpreted and responded to certain items differently, revealing partial metric invariance. Gender differences were particularly prominent for item 2 (“*How afraid are you of gaining 2 pounds?*) and item 5 (“*Do you ever feel fat?*), with both items being more strongly associated with the underlying weight concerns construct for women than for men. Brasil et al. [34] concluded that the WCS may underestimate weight concerns in men, as some items may be less relevant for males.

### The present study

Given the increased incidence of eating disorders among adolescents, early identification of those at-risk is essential in enabling adolescents to be linked to timely and appropriate evidence-based interventions that can reduce long-term harm. Apart from the principal components analysis conducted in 1994, the structure of the WCS has not been validated in adolescent samples and there are no studies examining its use in adolescent boys. Therefore, the present study aims to address this gap by examining the factor structure of the WCS in a large sample of adolescents, assessing gender invariance and the scale’s relationship with psychological measures of distress, wellbeing, self-harm and resilience. In doing so, we aim to determine whether the WCS is a psychometrically sound measure suitable for use as a screening tool in adolescents.

## Method

### Participants

Participants were adolescents (12–17 years) who took part in a large-scale evaluation of a mobile phone ban in South Australian schools [37]. Students from five secondary schools completed surveys between May and August 2023. The study was approved by the Flinders University Human Research Ethics Committee (Project ID: 5954) and the Department of Education and Child Development Ethics Committee, granting passive (opt-out) written parental consent prior to testing, and active (opt-in) written student consent on the day of the surveys. A detailed outline of the study procedures and data collection is provided in King et al., [37]. The protocol for this trial was preregistered in December 2023 (see: osf.io/pnjtk) prior to data analysis due to uncertainties regarding schools’ participation.

### Measures

#### Demographics

All participants were asked demographic questions relating to age, self-identified gender (male, female, non-binary or other) and residential postcode. Residential postcode was used to determine socio-economic status (SES) according to the 2021 Index of Relative Socioeconomic Disadvantage (IRSD) Socioeconomic Indexes for Areas (SEIFA) in Australia (Australian Bureau of Statistics, [38]). Scores are standardised to a distribution with a mean of 1000 and standard deviation of 100, with higher scores indicating higher socio-economic advantage.

#### Weight concerns scale

Eating disorder risk was assessed using the Weight Concern Scale (WCS; [24, 39]), which comprises of items scored on 4-point (Item 4), 5-point (Items 1, 2 and 5) and 7-point (Item 3) Likert scales, where scores range from 0 to 100. Table 1 provides a summary of each item with its respective Likert scale scoring and descriptive statistics. Internal consistency for the WCS in the present study was ω = 0.82.

**Table 1:** Weight Concerns Scale: items, Likert scale descriptors and descriptive statistics; unshaded lines female (*n* = 529), light grey shaded male (*n* = 493), darker grey shaded all (*n* = 1022)

Item Number	Item Content	Likert Scale	M (SD; min-max)	Skewness (SE)	Kurtosis (SE)
WCS1	*How much more or less do you feel you worry about your weight and body shape than other people of the same gender and age?*	1 = A lot less than others 2 = A little less than others 3 = About the same as others 4 = A little more than others 5 = A lot more than others	3.08 (1.33; 1–5)	− 0.21 (0.11)	−1.05 (0.21)
			2.36 (1.34; 1–5)	0.49 (0.11)	−1.06 (0.22)
			2.73 (1.38; 1–5)	0.11 (0.08)	−1.25 (0.15)
WCS2	*How afraid are you of gaining 1.5 kg?*	1 = Not afraid 2 = Slightly afraid 3 = Moderately afraid 4 = Very afraid 5 = Terrified	2.57 (1.38; 1–5)	− 0.21 (0.11)	−1.10 (0.21)
			1.63 (1.08; 1–5)	1.74 (0.11)	2.13 (0.22)
			2.12 (1.33; 1–5)	0.88 (0.08)	− 0.49 (0.15)
WCS3	*When was the last time you went on a diet?*	1 = Never 2 = About 1 year ago 3 = About 6 months ago 4 = About 3 months ago 5 = About 1 month ago 6 = Less than 1 month ago 7 = I’m now on a diet	2.57 (2.23; 1–7)	1.08 (0.11)	− 0.49 (0.21)
			2.10 (2.01; 1–7)	1.62 (0.11)	1.04 (0.22)
			2.34 (2.14; 1–7)	1.31 (0.08)	0.09 (0.15)
WCS4	*Compared to other things*,* how important is your weight to you?*	1 = Not important compared to other things in my life 2 = A little more important than some other things in my life 3 = More important than most, but not all things in my life 4 = Most important thing in my life	2.04 (0.90; 1–4)	0.47 (0.11)	− 0.66 (0.21)
			1.75 (0.81; 1–4)	0.83 (0.11)	0.01 (0.22)
			1.90 (0.87; 1–4)	0.64 (0.08)	− 0.41 (0.15)
WCS5	*Do you ever feel fat?*	1 = Never 2 = Rarely 3 = Sometimes 4 = Often 5 = Always	2.86 (1.37; 1–5)	0.13 (0.11)	−1.18 (0.21)
			1.93 (1.21; 1–5)	1.18 (0.11)	0.34 (0.22)
			2.41 (0.56; 1–5)	0.56 (0.08)	− 0.96 (0.15)
WCS Total	WCS1-WCS5		39.71 (27.90; 0–100)	0.44 (0.11)	− 0.79 (0.21)
			23.27 (21.61; 0–100)	0.99 (0.11)	0.34 (0.22)
			31.80 (26.36; 0–100)	0.74 (0.08)	− 0.34 (0.15)

#### Psychological distress

The Kessler Psychological Distress Scale (K10; [40]), is a brief 10-question symptom scale that measures non-specific psychological distress, focusing on emotional states and symptoms associated with anxiety (i.e., agitation, nervousness) and depression (i.e., negative affect, fatigue) over the past month. The K10 is a valid screening measure in Australian contexts, with a strong relationship between high scores on the K10 and a diagnosis of a psychological disorder [41, 42]. The K10 has utility for assessing psychological distress in health care and epidemiological research [43–45] and was scored using the Australian Bureau of Statistics categories (10–15 = low; 16–21 = moderate; 22–29 = high; 30–50 = very high), with higher scores reflecting higher psychological distress. The internal consistency of the scale was ω = 0.91.

#### Life satisfaction

Life satisfaction was assessed using the Programme for International Student Assessment (PISA) study’s 3-item measure of life satisfaction, which surveys life purpose or meaning. All three items are self-referential statements and include: “*My life has clear meaning or purpose*”, “ *I have discovered a satisfactory meaning in life*” and “*I have a clear sense of what gives meaning to my life*.” Items are assessed on a 4-point Likert scale ranging from “strongly disagree” to “strongly agree”, with higher scores indicating higher life satisfaction. The internal consistency for the 3-item life satisfaction scale in the present study was ω = 0.89.

#### Resilience

Resilience was assess using the PISA’s study 5-item measure of resilience, which surveys one’s ability to adapt to challenges and overcome difficulties. Sample items include: “*I usually manage one way or another*” and “*I feel that I can handle many things at a time*”. Items are rated on a 4-point Likert scale ranging from “strongly disagree” to “strongly agree”, with higher scores indicating higher resilience. Its internal consistency in the present study was ω = 0.83.

#### Self-injurious thoughts and behaviour

Frequency of past-month self-injurious thought was assessed using a single item from the Self Injurious Thoughts and Behaviours Interview-Revised (SITBI-R; [46]): “*On how many days over the past month have you had thoughts about purposely hurting yourself without wanting to die?*”. This item was rated on a 7-point Likert scale: 0 = No days; 1 = 1–5 days; 2 = 6–12 days; 3 = 13–15 days; 4 = 16–22 days; 5 = 23–27 days; 6 = Every day, with higher scores reflecting higher frequency of self-injurious thoughts.

Similarly, the frequency of past-month self-injurious behaviours was assessed using a further item from the SITBI-R: frequency of SIB in the past month, our main outcome, was assessed by a further item from the SITBI-R: “*How many times in the past month have you purposely hurt yourself without wanting to die (e.g. cut*,* or carved skin*,* hit yourself on purpose*,* burned your skin*,* bit yourself*,* inserted objects into your skin or nails)?*”. This single-item was measured on a 7-point Likert scale: 0 = No times; 1 = 1–5 times; 2 = 6–12 times; 3 = 13–15 times; 4 = 16–22 times; 5 = 23–27 times; 6 = 30+ times, with higher scores reflecting higher frequency of self-harm behaviours.

### Statistical analyses

Confirmatory Factor Analysis (CFA) was performed with MPlus software version 8.10, using weighted least squares with mean and variance adjustment (WLSMV), as recommended for use with categorical data [47]. Model fit was assessed using the chi-square test of exact fit, as recommended [48], alongside approximate fit indices: Root-Mean-Square Error of Approximation (RMSEA), Comparative Fit Index (CFI), and Tucker-Lewis Index (TLI). Following established guidelines [48, 49], RMSEA values < 0.06 were interpreted as indicating good fit, values between 0.06 and 0.08 as acceptable, and values > 0.10 as poor fit. CFI and TLI values ≥ 0.95 were considered indicative of excellent fit, while values ≥ 0.90 suggested adequate fit. However, it has been noted that RMSEA can be artificially large in models with small degrees of freedom and sample size and are thus interpreted with caution [50].

Factor invariance was evaluated across gender (male versus female) and socio-economic-status (SEIFA < 1000 versus SEIFA > 1000) by testing three models: Configural Invariance, Metric Invariance, and Scalar Invariance. The Configural Invariance Model estimates separate factor loadings and item threshold values (cut points between the ordinal responses) for each item between the two groups, representing the “baseline” model against which the subsequent two models are compared. The Metric Invariance Model fixes the factor loadings for each item to be equivalent across the two groups but allows the item thresholds to differ. The Scalar Invariance Model fixes both the factor loadings and item threshold values between the two groups. If the chi-square difference value between the models is significant, it indicates that constraining the parameters of the nested model significantly worsens the fit of the model, which indicates measurement non-invariance. If the chi-square difference value is not significant this indicates that constraining the parameters of the nested model did not significantly worsen the fit of the model, indicating measurement invariance of the parameters constrained to be equal in the nested model.

All remaining analyses were conducted using the Statistical Package for Social Sciences, Version 30 (SPSS; IBM SPSS, [51]). Logistic regression analyses were conducted to explore a WCS cut-off score of ≥ 47 [26] and its associations with our psychological outcome variables. Pearson correlations were performed to assess divergent validity using our measures of psychological distress (K10), life satisfaction, resilience and self-harm.

## Results

### Sample characteristics and missing data analysis

A total of 1169 participants completed the WCS measure. Participants in the total sample had a mean age of 14.68 (*SD* = 1.55), with 47.9% self-identifying as female and a mean SES score of 1000.01 (*SD* = 130.96). Closer inspection of the data revealed a total of 147 participants had missing data for either gender or SES, thus precluding our ability to include them in invariance testing analyses. To investigate whether data were missing at random, logistic regression analyses were conducted to examine baseline predictors of missingness. Results are presented in Table 2, highlighting no significant differences between the two groups on any of the baseline predictors. Therefore, participants with missing data on these demographic variables were removed from the final sample, with all further analyses comprising of a final sample of 1022 participants. Table 3 provides demographic and baseline characteristics of both the total (*n* = 1169) and final samples (*n* = 1022). No differences were found between the two groups in any of the parameters assessed.

**Table 2:** Logistic regression analyses investigating predictors of missingness between participants without missing data for gender and SES (*n* = 1022; coded as 0) and those with missing data (*n* = 147; coded as 1)

Outcome	Odds Ratio (95% CI), *p*
Weight Concerns Scale	1.01 (0.99, 1.02), *0.29*
Psychological Distress	1.02 (0.98, 1.07), *0.38*
Life Satisfaction	0.86 (0.52, 1.42), *0.55*
Resilience	0.90 (0.47, 1.72), *0.75*
Self-injurious thoughts	0.84 (0.65, 1.07), *0.16*
Self-injurious behaviours	1.04 (0.81, 1.02), *0.75*

**Table 3:** Demographic characteristics and baseline descriptive statistics for participants in the total sample (*n* = 1169) and participants in the final sample (*n* = 1022)

Variable	Total Sample (*n* = 1169)	Final Sample (*n* = 1022)
Age (M, SD)	14.68 (1.55)	14.73 (1.55)
Gender (%)		
*Male* *Female* *Non-binary* *Other*	46.8% 47.9% 2.1% 3.2%	46.8% 47.9% 2.1% 3.2%
SES score	1000.01 (130.96)	999.71 (133.41)
Weight Concerns (M, SD)	32.51 (26.87)	31.80 (26.36)
Psychological Distress (M, SD)	23.29 (8.90)	22.99 (8.65)
Life Satisfaction (M, SD)	2.87 (0.74)	2.91 (0.70)
Resilience (M, SD)	2.99 (0.58)	3.01 (0.54)
Past-month self-injurious thoughts (%)		
*No days* *1–5 days* *6–12 days* *13–15 days* *16–22 days* *23–27 days* *Every day*	77.1% 12.3% 2.0% 2.0% 1.5% 1.0% 4.1%	77.1% 12.3% 2.0% 2.0% 1.5% 1.0% 4.1%
Past-month self-injurious behaviours (%)		
*No times* *1–5 times* *6–12 times* *13–15 times* *16–22 times* *23–27 times* 30 + times	7.61% 5.73% 1.54% 0.77% 0.60% 0.43% 1.37%	6.26% 5.77% 1.66% 0.78% 0.68% 0.39% 0.78%

### Confirmatory factor analysis and invariance testing

Confirmatory factor analysis results replicated the original single-factor WCS structure in our large adolescent sample. The one-factor model demonstrated mixed model fit: χ²(5) = 44.83, *p* <.001, RMSEA = 0.09, CFI = 0.99. RMSEA suggested poor fit according to conventional model fit indices (Kline, 2016), however CFI and TLI values indicate excellent fit. Given the small number of degrees of freedom, RMSEA values may be inflated. Goodness-of-fit statistics and factor loadings for the whole sample and across gender (male versus female) are presented in Table 4. For the whole sample, factor loadings were acceptable (> 0.70) across all items apart from Item 3: “*When was the last time you went on a diet?*”, which had a factor loading of 0.621 (λ = 0.507 in males and λ = 0.671 in females). In addition, Item 4: “*Compared to other things*,* how important is your weight to you?”* had a factor loading < 0.70 for males (λ = 0.647).

**Table 4:** Confirmatory factor analysis: goodness-of-fit statistics and factor loadings for the Weight Concerns Scale

Goodness-of-fit statistic	Model fit indices
RMSEA	0.09
CFI	0.99
TLI	0.99
χ^2^ (*df*)	44.83 (*5*)*
Whole sample (*n* = 1022)	
Item number	**Factor loadings (λ)**
WCS1	0.818
WCS2	0.883
WCS3	0.621
WCS4	0.757
WCS5	0.883
Females (*n* = 529)	
Item number	**Factor loadings (λ)**
WCS1	0.861
WCS2	0.884
WCS3	0.671
WCS4	0.825
WCS5	0.906
Males (*n* = 493)	
Item number	**Factor loadings (λ)**
WCS1	0.704
WCS2	0.859
WCS3	0.507
WCS4	0.647
WCS5	0.823

*Denotes significance at *p* <.05; *RMSEA* Root mean square error of approximation, *CFI* Comparative Fit Index, *TLI* Tucker-Lewis Index, *WCS* Weight Concerns Scale

Invariance testing was conducted by gender (female versus male) and SES (below versus above SEIFA mean of 1000). Table 5 presents the results of invariance testing. Measurement invariance was unable to provide a Metric model when labelling variables as categorical, thus invariance testing was conducted with the variable coded as continuous to obtain statistics for the three models (Configural, Metric and Scalar). Across both invariance testing models, there were significant chi-square value differences between the Configural, Metric and Scalar models, thus supporting gender and SES non-invariance. Therefore, factor loadings cannot be constrained to be equal across any of our moderating groups, and item thresholds are significantly different across the two groups.

**Table 5:** Measurement Invariance Testing Results for Weight Concerns Scale Across Gender and Socio-Economic-Status (SES)

Model	No. of parameters	Chi-square (df)	Models compared	Chi-square (df)
Gender: female (*N* = 529) versus male (*N* = 493)				
Configural	30	51.43 (10)*	Configural vs. Metric	6.54 (4)
Metric	26	57.98 (14)*	Configural vs. Scalar	49.96 (8)*
Scalar	22	101.39 (18)*	Metric vs. Scalar	43.41 (4)*
SES: low (***N*** = 383) versus high (***N*** = 639)				
Configural	30	47.14 (10)*	Configural vs. Metric	16.49 (4)*
Metric	26	63.62 (14)*	Configural vs. Scalar	32.41 (8)*
Scalar	22	79.54 (18)*	Metric vs. Scalar	15.92 (4)*

*denotes significance at *p* <.05

### Validity analyses

The descriptive statistics and internal consistency of the WCS, as well as all other included measures, are reported in Table 6 for these four groups (male lower SES, male higher SES, female lower SES, female higher SES). Logistic regression analyses were conducted to explore whether a WCS cut-off score ≥ 47 was associated with our psychological outcome variables across our four groups. Results are presented in Table 7. Findings revealed a cut-off score of ≥ 47 was significantly associated with psychological distress across all samples, apart from males from lower SES backgrounds, the only group to not emerge with any significant associations. Moreover, a WCS cut-off score of ≥ 47 was associated with lower resilience and higher frequency of self-harm thoughts in females from higher SES backgrounds. Pearson correlations were used to assess divergent validity between the WCS and our outcome measures, including psychological distress, life satisfaction, resilience and self-injurious thoughts and behaviours across our four groups. Results are presented in Supplementary Table 1 for females (higher and lower SES) and for males (higher and lower SES) in Supplementary Table 2. All correlations between the WCS and our psychological outcomes were in the expected directions, with positive correlations with psychological distress, self-injurious thoughts and behaviours and negative correlations with life satisfaction and resilience.

**Table 6:** Descriptive statistics (means and standard deviations) and Internal Consistency (McDonald’s Omega) for all outcome measures across our four groups

Female lower SES (*n* = 226)						
	*N*	Mean (SD)		Range	Cronbach’s alpha	McDonald’s Omega
Weight Concerns	226	39.23 (26.36)	0–100		0.82	0.83
Psychological distress	226	25.65 (8.96)	10–50		0.91	0.91
Life satisfaction	226	2.82 (0.68)	1–4		0.89	0.89
Resilience	226	2.94 (0.54)	1–4		0.82	0.83
Past-month self-injurious thoughts	223	0.65 (1.58)	0–6		-	-
Past-month self-injurious behaviours	43	1.44 (1.62)	0–6		-	-
Female higher SES (*n* = 303)						
Weight Concerns	303	40.06 (29.02)	0–100		0.86	0.87
Psychological distress	303	24.78 (8.44)	10–48		0.90	0.90
Life satisfaction	303	2.82 (0.68)	1–4		0.85	0.85
Resilience	303	2.96 (0.53)	1–4		0.81	0.82
Past-month self-injurious thoughts	303	0.68 (1.42)	0–6		-	-
Past-month self-injurious behaviours	59	1.20 (1.34)	0–6		-	-
Male lower SES (*n* = 157)						
Weight Concerns	157	20.80 (21.45)	0–90		0.73	0.74
Psychological distress	157	19.80 (6.83)	10–46		0.84	0.83
Life satisfaction	157	3.05 (0.69)	1–4		0.91	0.91
Resilience	157	3.09 (0.45)	1.80–4		0.76	0.75
Past-month self-injurious thoughts	155	0.24 (0.87)	0–6		-	-
Past-month self-injurious behaviours	14	1.00 (1.62)	0–6		-	-
Male higher SES (*n* = 336)						
Weight Concerns	336	24.43 (21.62)	0–100		0.70	0.70
Psychological distress	336	21.08 (8.49)	10–50		0.92	0.91
Life satisfaction	336	2.98 (0.72)	1–4		0.91	0.91
Resilience	336	3.08 (0.57)	1–4		0.86	0.87
Past-month self-injurious thoughts	333	40 (1.22)	0–6		-	-
Past-month self-injurious behaviours	51	1.29 (1.88)	0–6		-	-

**Table 7:** Logistic regression analyses exploring WCS cut-off score of ≥ 47 on predicting differences in our psychological outcome measures across our four adolescent groups

Outcome	Odds Ratio (95% CI), *p*
Female lower SES (*n* = 226)	
Psychological Distress	0.09 (1.05, 1.14), < 0.01*
Life Satisfaction	− 0.37 (0.40, 1.21), *0.20*
Resilience	− 0.24 (0.39, 1.58), *0.51*
Self-injurious thoughts	0.42 (0.87, 2.68), *0.14*
Self-injurious behaviours	0.23 (0.52, 3.01), *0.61*
Female higher SES (*n* = 303)	
Psychological Distress	0.08 (1.05, 1.13), < 0.01*
Life Satisfaction	− 0.12 (0.56, 1.41), *0.60*
Resilience	− 0.58 (0.32, 0.99), 0.05*
Self-injurious thoughts	0.51 (1.10, 2.54), 0.02*
Self-injurious behaviours	− 0.05 (0.49, 1.84), *0.88*
Male lower SES (*n *= 157)	
Psychological Distress	0.06 (0.99, 1.14), *0.09*
Life Satisfaction	0.24 (0.59, 2.75), *0.54*
Resilience	− 0.35 (0.22, 2.25), *0.56*
Self-injurious thoughts	0.24 (0.86, 1.89), *0.24*
Self-injurious behaviours	0.01 (0.38, 2.61), *0.99*
Male higher SES (*n* = 336)	
Psychological Distress	0.10 (1.06, 1.15), < 0.01*
Life Satisfaction	0.07 (0.63, 1.83), *0.81*
Resilience	0.36 (0.70, 2.92), *0.33*
Self-injurious thoughts	− 0.01 (0.62, 1.61), *0.99*
Self-injurious behaviours	0.11 (0.63, 1.96), *0.71*

* indicates significance at *p* <.05

## Discussion

The present study reports on the factor structure and psychometric properties of the WCS in a large sample of Australian adolescents aged 12 to 17 years, with the objective of determining the appropriateness of this scale and its potential as a first-step screening and early detection tool for adolescents. To this end, we examined the model fit of the WCS in our sample and assessed measurement invariance by gender (male versus female) and socio-economic-status (lower versus higher). First, we replicated the original WCS one-factor structure, with model fit indices in our adolescent sample (χ²[5] = 44.83, *p* <.001, RMSEA = 0.09, CFI = 0.99. RMSEA) comparable to other previous validations of the WCS in female university students [35, 36]. Our findings provide support for the structural validity of this measure for use with adolescents.

Second, to our knowledge, the present study is the first to confirm the factor structure of the WCS in adolescents, but also socio-economic-status and gender invariance in this population. Previous research on the WCS has examined gender invariance of this scale with a university sample only [34]. Our findings revealed gender and socio-economic-status non-invariance, suggesting significant differences in how the WCS functions across different gender and socio-economic-status backgrounds. This suggests that further examination of the WCS across different genders and the socio-economic-status spectrum is required. One item requiring revision is Item 3, rated on a 7-point Likert scale in contrast to the other 4- or 5-point scales used for the remaining items. In our samples, factor loadings for Item 3 ranged from.51 −.67. The use of mixed Likert scale response formats within this measure may introduce measurement error and reduce the comparability of item responses, which may impact on the scale’s reliability and validity. Future research should consider adaptations to Item 3 and standardisation of response formats to a 5-point Likert scale. Moreover, item 4 (*how important is your weight to you?”*) may require rewording to improve its suitability for males.

Third, we explored associations of the WCS cut-off score with our psychological outcome measures across the four adolescent groups. A WCS cut-off score ≥ 47 was significantly associated with psychological distress across all samples, but not in males from lower SES backgrounds. This was the only group in which a WCS cut-off score was not significantly associated with any of the psychological outcome measures, thus suggesting further work is needed to ensure the WCS is appropriate for this cohort. Moreover, for females from higher SES, a WCS cut-off score ≥ 47 was also significantly associated with lower resilience scores and higher frequency of self-harm thoughts. While our findings indicate the potential utility of the WCS in identifying adolescents at-risk, further research is needed to improve the appropriateness of this measure in adolescent boys and to establish convergent validity of the WCS with established measures of eating disorders, such as the Eating Disorder Examination Questionnaire, as well as broader psychological outcomes highly comorbid with eating disorders such as anxiety, depression and suicidal thoughts.

While our findings provide support for the original WCS factor structure in adolescents, they also highlight the potential for expanding its application beyond its conventional use and using it as a screening tool. Compared to the ADO-BED and the SCOFF, the WCS has the great advantage of not being tied into a diagnosis, thus capturing the shifting eating disorder psychopathology that is present in adolescents and making it suitable for early detection before a diagnosis emerges. Conceptually, the WCS captures early cognitions and attitudes towards weight and dieting, which are indicative of increased disordered eating risk. In contrast, behavioural screeners such as the SCOFF identify the presence of more overt eating disorder behaviours (i.e., vomiting). These tools may therefore compliment each other across different stages of prevention, early intervention and in informing subsequent clinical assessment and diagnosis. Future studies should explore a stepped-screening approach, whereby brief disordered eating risk screening (i.e., WCS) is used as an initial first step for early identification prior to the implementation of more behaviourally focused screening among at-risk individuals.

Several limitations should be considered when interpreting our findings. First, due to the absence of eating disorder measures in our study, we were unable to test convergent validity of the WCS with established ED measures. Similarly, the lack of diagnostic interview data precluded evaluation of the WCS in relation to clinical diagnostic status. Second, the present study did not include psychological outcome measures of comorbidities associated with EDs, such as depression anxiety and suicidal ideation, which may further inform construct validity and clinical utility. Third, our sample was derived from a single cultural context (Australian adolescents) and factor structure, and cut-off scores may differ across different cultural settings. Lastly, as the present study focused on the factor structure and gender invariance of the WCS, screening performance indices (i.e., sensitivity, specificity and optimal cut-off scores) were not examined and should be established in future research.

Considering these limitations, there remains, however, many gaps in our knowledge to address in future research prior to exploring the status of the WCS as a screening tool. Future research needs to establish validity of the WCS in adolescents as well as its associations with ED psychopathology and behaviours across diverse populations. In addition, validation against diagnostic interview is crucial to establishing the WCS performance as a screening tool. Moreover, another important avenue for future research is in establishing the potential harm and benefits associated with universal screening of EDs among adolescents. There is currently a lack of evidence from trials comparing routine screening versus no screening [19], meaning that the balance of benefits and harms associated with universal screening remains uncertain, which highlights a significant gap in the research evidence. Future research should also focus on further refining item weighting and scoring. This research should engage in a co-design process with adolescents to adjust wording and scoring points for all items, which may address the issue of non-invariance. For example, the adapted version of the WCS used by Field and colleagues [52] had wording equally relevant to all genders. Given that males are more likely than females to focus on wanting to increase muscularity rather than being thin [53], Field and colleagues [52] adapted questions to ensure their appropriateness for males. Specifically, questions on thinness were replaced with questions about the importance of not being fat or the desire to not be fat. Example questions include: “*In the past year*,* how often have you felt fat?”* and “*In the past year how often have you desired to not to be fat?*”. Such adaptations highlight the importance of ensuring that screening tool for disorder eating in adolescents are developmentally and gender appropriate for diverse populations.

In conclusion, the present study replicated the original one-factor structure of the WCS in a large sample of Australian adolescents, aged 12 to 17 years. Invariance testing revealed socio-economic-status and gender non-invariance, suggesting the WCS scale functions differently across these subgroups. The present study was the first to examine the factor structure of the WCS in adolescents and the first to consider its use as a screening tool. Our findings highlight its potential as a screening tool. Adapting the wording of some of the WCS items for further examination in terms of its association with disordered eating is an important step in the identification of a screening tool that can be used across adolescents. If proved suitable, this would represent a significant contribution to the early detection of EDs in adolescents. With the increased incidence of eating disorders among youths, it is crucial to have robust screening tools that can detect disordered eating and link young people into evidence-based treatment as early as possible.

## Supplementary Information

Below is the link to the electronic supplementary material.

ESM 1 DOCX (29.3 KB)

## Data Availability

The data that support the findings of this study are available from the corresponding author upon reasonable request.
