# Supplementary material for: The weight concerns scale in Australian adolescents: Factor structure, gender invariance, and screening utility
Source: Eur Child Adolesc Psychiatry. 2026 Jun 22;35(10):3313–23. doi: 10.1007/s00787-026-03101-8 (PMC13696600; doi:10.1007/s00787-026-03101-8)
Supplement: Supplementary file 1 [file 787_2026_3101_MOESM1_ESM.docx]

**Supplementary Table 1.** Pearson Correlations for Females (lower SES in lower diagonal, higher SES in upper diagonal)

|  | **1** | **2** | **3** | **4** | **5** | **6** |
| --- | --- | --- | --- | --- | --- | --- |
| 1. WCS |  | .44* | -.32* | -.26* | .40* | .36* |
| 2. K10 | .46* |  | -.51* | -.39* | .44* | .45* |
| 3. Life satisfaction | -.34* | -.43* |  | .45* | -.36* | -.15 |
| 4. Resilience | -.32* | -.39* | .54* |  | -.29* | -.09 |
| 5. Self-harm thoughts | .40* | .55* | -.33* | -.30* |  | .65* |
| 6. Self-harm behaviours | .30 | .48* | -.28 | -.40* | .77* |  |

**Supplementary Table 2.** Pearson Correlations for Males (lower SES in lower diagonal, higher SES in upper diagonal)

|  | **1** | **2** | **3** | **4** | **5** | **6** |
| --- | --- | --- | --- | --- | --- | --- |
| 1. WCS |  | .35* | -.10 | -.12* | .16* | .29* |
| 2. K10 | .19* |  | -.38* | -.47* | .39* | .32* |
| 3. Life satisfaction | -.14 | -.41* |  | .49* | -.31* | .08 |
| 4. Resilience | -.14 | -.36* | .47* |  | -.32* | -.21 |
| 5. Self-harm thoughts | .08 | .47* | -.36* | -.21* |  | .74* |
| 6. Self-harm behaviours | -.05 | -.27 | .19 | .11 | -.12 |  |
